# A qualitative study investigating the meaning of participation to improve the measurement of this construct

**DOI:** 10.1007/s11136-019-02179-9

**Published:** 2019-04-16

**Authors:** Astrid de Wind, Allard J. van der Beek, Edwin J. Boezeman, Rosalie Swenneker, Johannes R. Anema, Angela G. E. M. de Boer, Heleen Beckerman, Jan L. Hoving, Karen Nieuwenhuijsen, Micky Scharn, Mariska Stam, Caroline B. Terwee, Monique H. W. Frings-Dresen, Sietske J. Tamminga

**Affiliations:** 1 Department of Public and Occupational Health, Amsterdam Public Health Research Institute, Amsterdam UMC, VU University, Van der Boechorststraat 7, 1081 BT Amsterdam, The Netherlands; 2 Coronel Institute of Occupational Health, Amsterdam Public Health Research Institute, Amsterdam UMC, AMC, Meibergdreef 9, 1105 AZ Amsterdam, The Netherlands; 3 Department of Rehabilitation Medicine, MS Center Amsterdam, Amsterdam Public Health Research Institute, Amsterdam UMC, VU University, Van der Boechorststraat 7, 1081 BT Amsterdam, The Netherlands; 4 Research Center for Insurance Medicine: collaboration between AMC–UMCG–UWV–VUmc, Amsterdam, The Netherlands; 5 Department of Otolaryngology–Head and Neck Surgery, Amsterdam Public Health Research Institute, Amsterdam UMC, VU University, Van der Boechorststraat 7, 1081 BT Amsterdam, The Netherlands; 6 Department of Epidemiology and Biostatistics, Amsterdam Public Health Research Institute, Amsterdam UMC, VU University, Van der Boechorststraat 7, 1081 BT Amsterdam, The Netherlands

**Keywords:** Participation, ICF, PROMIS, Measurement development, Item bank, Qualitative research

## Abstract

**Purpose:**

The purpose of this study was to improve the measurement of participation. Research questions were as follows: (1) What constitutes participation according to adults? (2) Do they mention participation subdomains that are not covered in the Patient-Reported Outcomes Measurement Information System (PROMIS) item bank “Ability to Participate in Social Roles and Activities”?

**Methods:**

Semi-structured interviews were conducted with 46 adults from the general population. Interviews were thematically analysed using the International Classification of Functioning, Disability and Health (ICF) as conceptual framework. Thereafter, assigned codes were compared to PROMIS item bank.

**Results:**

Participants mentioned a variety of participation subdomains that were meaningful to them, such as socializing and employment. All subdomains could be classified into the ICF. The following subdomains were not covered by the PROMIS item bank: acquisition of necessities, education life, economic life, community life, and religion and spirituality. Also a distinction between remunerative (i.e. paid) and non-remunerative (i.e. unpaid) employment, and domestic life was missing. Several ICF sub-codes were not mentioned, such as ceremonies.

**Conclusions:**

Many participation subdomains were mentioned to be meaningful. As several of these subdomains are not covered in the PROMIS item bank, it may benefit from extension with new (patient-)reported subdomains of participation.

## Introduction

There is increasing awareness that participation is important to an inclusive society, including people with and without disabilities, and their families [1]. Participation is also considered a determinant of health [2]. However, the ability to participate in social roles and activities is difficult to measure as it involves a diversity of subdomains, while not all subdomains are applicable to everyone. Moreover, it has been suggested that people with disabilities perceive participation as a dynamic process involving negotiation and balancing of competing needs and values across individual, social, and societal levels, which constitute ‘push’ and ‘pull’ factors on people’s ability to participate in ways that they find meaningful and satisfying [3]. Eyssen et al. systematically reviewed participation measurement instruments. They concluded that most instruments aiming to measure participation only do so to a limited extent [4]. Many instruments seemed to measure either other related constructs, such as activity level, or they measure only one subdomain of participation (e.g. work, social, or home) [4]. This might not be optimal due to the dynamic nature of participation, as well as the fact that participation is not the same for everyone. Hammel et al. recommended that ‘any effort to measure objective participation would have to be flexible enough to deal with the fact that different people will need, desire and endorse different aspects of participation and that very different patterns of participation can still reflect full participation’ [3].

A promising alternative for existing measurement instruments on participation, which addresses Hammel’s recommendation, is the PROMIS v2.0 item bank “Ability to Participate in Social Roles and Activities” of the Patient-Reported Outcomes Measurement Information System (PROMIS^®^) [5, 6]. This PROMIS item bank allows for being administered as a personalized short form or through Computer Adaptive Testing, and as such, it allows for only using a selected number of automatically generated items that are informative to each specific person [6, 7]. As the measurement is based on Item Response Theory (IRT), people can still be compared with each other. PROMIS is a generic assessment system for the measurement of patient-reported health. It is an initiative started by six US research centres and the US National Institutes of Health aiming to help health professionals with the standardized measurement of health(-related issues) [5]. PROMIS adopted the perspective of the World Health Organization (WHO) that three general health domains can be distinguished, i.e. physical, mental, and social health [8], but proceeded to specify specific health domains [4, 9].

Researchers have argued that the ICF conceptual framework and the PROMIS conceptual framework both categorize health domains relevant for measurement among patients [9], that they are complementary to each other, but that have their own unique features too [9]. Both assume that health problems may undermine functioning of individuals and hinder their ability to partake in activities and fulfil social roles [8]. Furthermore, the activities and roles of individuals may also be detrimental to their health according to both perspectives [8, 9]. The ICF framework makes clear that health may affect activities and participation of individuals [8]. Additionally, PROMIS discusses the ability to participate in social roles and activities as an indicator of social health of individuals [5].

Although the concepts of participation within the ICF conceptual framework and PROMIS are overlapping, previous research found that the subcategories of ICF that belong to the concepts “activities and participation” appear to be more exhaustive than the subcategories of the PROMIS item bank “Ability to Participate in Social Roles and Activities” [9]. It is unclear whether adults nowadays consider these additional subcategories as constituting to their participation. It would, thus, be worthwhile to study what adults constitute to their participation and to compare this to ICF. This knowledge would help us to better understand whether the item bank may benefit from extension with other items.

Therefore, the purpose of this study is to improve measurement of participation. Our research questions are as follows: (1) What constitutes participation according to adults? (2) Do they mention subdomains of participation that are not covered in the PROMIS item bank “Ability to Participate in Social Roles and Activities”?

## Materials and methods

This study consisted of two parts. Part 1 addresses research question 1 by conducting and analysing semi-structured interviews with adults. Part 2 addresses research question 2 by comparing assigned codes to the PROMIS item bank “Ability to Participate in Social Roles and Activities”. The Medical Ethical Committee of VU University Medical Center (Amsterdam, the Netherlands) declared that the Medical Research Involving Human Subjects Act does not apply to this study, and had no objection to execution of this study (reference number 2017.328). We used the items of the Consolidated criteria for reporting qualitative research (COREQ) for improving the quality of reporting qualitative research [10].

### Part 1: What constitutes participation to adults?

#### Study sample and recruitment

The only inclusion criterion was being 18 years or older. Not being able to have a conversation in Dutch was an exclusion criterion. Recruitment of participants took place via an invitation that was spread to an online panel of the Netherlands Patient Federation consisting of more than 3000 adults with and without health problems. This panel consists of patients and caregivers with experience with the health care system. About 1600 adults positively responded to our invitation to participate in an interview. As the choice for the panel of the Netherlands Patient Federation may result in selection bias with respect to ability to participate in society and in order to capture a broad range of perspectives, 50 adults were purposefully selected. The purposeful selection was based on the following characteristics, which all of the co-authors agreed on before the selection took place: educational level, age, sex, presence (yes/no) of a health problem (mental and/or physical), and their meaning of participation. Information on the meaning of participation was derived from the answer on the following question that was included in the original invitation: what does participation mean to you? One of the authors (ST) screened the answers and selected participants based on their answers in order to include participants showing a diversity of answers. The 50 adults were invited by telephone. Forty-six of them were willing to participate in an interview. Two were not available for an interview because of personal circumstances and two did not participate because of miscellaneous reasons. Participants signed an informed consent form, before the interview took place.

#### Interview guide and procedure

Prior to the beginning of the study, a semi-structured interview guide was created by AdW and ST, which was not pilot tested. It consisted of four parts: (1) meaning of participation, (2) satisfaction with participation, (3) limitations with participation (e.g. due to one or more health problem(s)), and (4) experiences with regard to attention for participation by healthcare professionals. Only answers to the first three questions were analysed. Parts 2 and 3 were included to gain a more in-depth understanding of what participation means to the participants. Part 4 was not analysed because this part aimed at a topic that we wanted to address in another study. The interview guide provided direction to the interviewers to explore the reported answers in more detail. Participants were provided with a layman synonym of the word ‘participation’ without any further definition. We did provide a few examples (see “Appendix” Interview guide) and explained that different people might endorse different aspects of participation. We did that because there is no consensus regarding the definition of participation in the literature [3]. Therefore, the choice for a certain definition might have influenced their answers.

Three of the co-authors (AdW, ST, RS) and two research assistants independently conducted the telephone interviews using this interview guide. AdW and ST were post-doc researchers, while RS was a research assistant. All interviewers were doing research in the field of occupational health. All interviewers were familiar with interview techniques, such as clarification, paraphrasing, and summarizing. None of the interviewers represented a patient organization or profession. The interviewers did not establish a relationship with the participants before the interviews took place. Interviews were audio-recorded. All participants were interviewed once-only. Before the start of the telephonic interview, each participant was explained that the purpose of the study was to study the meaning of participation to improve its measurement. Additionally, the interviewer explained that the position of the interviewer was impartial and that information was handled confidential. During the telephonic interviews, no one else was present expect the spouse of one participant as that participant was not able to communicate without her.

#### Data management and analysis

The interviews were transcribed verbatim. Transcripts were not returned to participants for comments. In order to manage the interview data, the software package for qualitative analysis Atlas.ti was used [11]. Analyses of the interview data took place in three steps. First, thematic content analysis was conducted by four coders (AdW, ST, EB, and RS). The ICF conceptual framework was used to code interview data according to the second-level codes within the chapters “Domestic Life (D6)”, “Interpersonal Interactions and Relationships (D7)”, “Major Life Areas (D8)”, and “Community, Social and Civic Life (D9)”. Second, inductive coding techniques were planned in case a domain of participation could not be coded according to the ICF conceptual framework. However, during the analyses everything revealed to fit within the ICF conceptual framework, and therefore, no inductive coding techniques were needed. The four coders coded independently to discover meaningful subdomains of participation. Two coders coded half of the interviews independently and two other coders coded the other half of the interviews. Third, a consensus meeting was held among the four coders. Finally, a meeting was organized with all co-authors, i.e. experts on participation, to discuss the results, and enhance robustness of the findings.

### Part 2: Are subdomains mentioned by adults also covered by items in the PROMIS item bank “Ability to Participate in Social Roles and Activities”?

#### Item bank

Codes that were assigned to the interview data in part 1 were compared to the PROMIS item bank “Ability to Participate in Social Roles and Activities v2.0” [12]. This item bank consists of 35 items on one’s perceived ability to take part in one’s usual social roles and activities. Example items are: “I have trouble doing my regular daily work around the house” and “I have to limit the things I do for fun with others”. The item bank has been developed and validated in the US with finance of the National Institutes of Health. Subsequently, it has been translated to Dutch-Flemish by the Dutch-Flemish PROMIS group [13]. Validation studies in Dutch and Flemish patients undergoing rehabilitation and a sample of the general population found good psychometric properties [7, 14].

#### Analysis

ST and AdW independently classified the items of the PROMIS item bank “Ability to Participate in Social Roles and Activities” to the ICF chapters (D6-D9) and corresponding (sub-)codes mentioned by adults in the interviews. In case of disagreement, a consensus meeting was held among ST, AdW, and EB.

## Results

### Study sample

The purposeful sampling strategy resulted in a heterogeneous study sample regarding sex, age, educational level, presence (yes/no) of a health problem (mental and/or physical), and participation (Table 1). The sample included 25 male and 21 female respondents. Respondents were on average 57 years of age (range 19–85 years). Twelve respondents had a low level of education, 16 an intermediate level, and 18 a high level. Most participants reported to participate in society, only a few reported only to participate to a very limited extent. On average, interviews lasted 15 min (range 6–27 min). After about 40 interviews, data saturation occurred as no new participation subdomains were mentioned in the last six interviews.

**Table 1:** Characteristics of the study population (*N* = 46)

Characteristic	Category	*N*
Age in years (mean, range)	N.A.	57, 19–85
Sex	Male	25
	Female	21
Educational level	Low	12
	Intermediate	16
	High	18
Presence of a health problem (mental and/or physical)	No	12
	Yes	34
Employment status^a^	Remunerative employment (paid work)	12
	Non-remunerative employment (unpaid work)	16
	Retired	17
	Other, no employment	17

^a^Number does not add up to 46 as combinations were possible

#### Part 1: What constitutes participation according to adults?

Participants mentioned a great variety of participation subdomains. All could be classified into the ICF conceptual framework (Table 2).

**Table 2:** Overview of subdomains of participation that are meaningful to adults and whether these are covered by the PROMIS item bank “Ability to Participate in Social Roles and Activities”

ICF chapter	ICF code	ICF sub-codes	Mentioned by at least one participant (x = yes)	Covered in PROMIS item bank “Ability to Participate in Social Roles and Activities”
Domestic life (D6)	Acquisition of necessities (d610–d629)		x	
		Acquiring a place to live (d610)	x	
		Acquisition of goods and services (d620)	x	
		Acquisition of necessities, other specified and unspecified (d629)	x	
	Household tasks (d630–d649)		x	RP1
		Preparing meals (d630)		
		Doing housework (d640)		
		Household tasks, other specified and unspecified (d649)	x	
	Caring for household objects and assisting others (d650–d669)		x	
		Caring for household objects (d650)	x	
		Assisting others (d660)	x	RP6 SRPPER05_CaPS SRPPER07_CaPS SRPPER08_CaPS SRPPER35_CaPS SRPPER54_CaPS
		Caring for household objects and assisting others, other specified and unspecified (d669)		
	Domestic life, other specified and unspecified (d698–d699)			SRPPR_CaSP1 SRPPER02 r1 SRPPER06_CaPS SRPPER09_CaPS SRPPER14 r1 SRPPER16 r1 SRPPER17 r1 SRPPER18_CaPS SRPPER22_CaPS SRPPER23_CaPS SRPPER26_CaPS SRPPER31_CaPS SRPPER37_CaPS SRPPER47_CaPS
Interpersonal interactions and relationships (D7)	General interpersonal interaction (d710–d729)		x	SRPPER43 r1
		Basic interpersonal interaction (d710)	x	
		Complex interpersonal interaction (d720)	x	
		General interpersonal interactions, other specified and unspecified (d729)		
	Particular interpersonal relationships(d730–d779)		x	
		Relating with strangers (d730)	x	
		Formal relationships (d740)	x	
		Informal social relationships (d750)	x	RP6 SRPPER20_CaPS SRPPER35_CaPS SRPPER36_CaPS SRPPER42 r1 SRPPER46_CaPS SRPPER54_CaPS SRPPER55 r1
		Family relationships (d760)	x	SRPPR_CaSP5 SRPPER01 r1 SRPPER07_CaPS SRPPER08_CaPS SRPPER17 r1
		Intimate relationships (d770)	x	
		Particular interpersonal relationships, other specified and unspecified (d779)		
	Interpersonal interactions and relationships, other specified and unspecified (d798–d799)		x	
Major life areas (D8)	Education life (d810–d839)		x	
		Informal education (d810)		
		Preschool education (d815)		
		School education (d820)		
		Vocational training (d825)		
		higher education (d830)		
		other education, specified and unspecified (d839)		
	Work and employment (d840–d859)		x	SRPPER02 r1 SRPPER06_CaPS SRPPER09_CaPS SRPPER16 r1 SRPPER23_CaPS SRPPER26_CaPS SRPPER37_CaPS SRPPER47_CaPS
		Apprenticeship (d840)	x	
		Acquiring, keeping, and terminating a job (d845)		
		Remunerative employment (d850)	x	
		Non-remunerative employment (d855)	x	
		Work and employment, other specified and unspecified (d859)		
	Economic life (d860–d879)		x	
		Basic economic transactions (d860)	x	
		Complex economic transactions (d865)		
		Economic self-sufficiency (d870)	x	
		Economic life, other specified and unspecified (d879)	x	
	Major life areas, other specified and unspecified (d989–d899)			SRPPER31_CaPS
Community, social and civic life (D9)	Community life (d910)		x	
		Informal associations (d9100)	x	
		Formal associations (d9101)	x	
		Ceremonies (d9102)		
		Community life, other specified and unspecified (d9108-d9109)	x	
	Recreation and leisure (d920)		x	SRPPER04_CaPS SRPPER11_CaPS
		Play (d9200)	x	
		Sports (d9201)	x	
		Arts and culture (d9202)	x	
		Crafts (d9203)		
		Hobbies (d9204)	x	
		Socializing (d9205)	x	SRPPR_CaSP1 SRPPER03 r1 SRPPER11_CaPS SRPPER13_CaPS SRPPER15_CaPS SRPPER21_CaPS SRPPER28 r1 SRPPER55 r1
		Recreation and leisure, other specified and unspecified (d9209)	x	
	Religion and spirituality (d930)		x	
		Human rights (d940)		
		Political life and citizenship (d950)	x	
	Community, social and civic life, other specified and unspecified (d998–d999)		x	

##### ICF chapter domestic life

Within the ICF chapter “Domestic Life”, all ICF codes (and most sub-codes) were mentioned, i.e. acquisition of necessities (d610–d629), household tasks (d630–d649), and caring for household objects and assisting others (d650–d669) (Table 2). Someone explained that having a place to live was an important aspect of participation for him:

“And, well, honestly I have to say, right now I do have a home again. I’ve been homeless for a while and to me that means that I am participating in society again.[…] Well, it means anyway that I’ll take care of myself again. That you’re part of society.” [1013, female, 39 years]

Several household tasks were mentioned as important aspects of participation, such as shopping and cleaning. The following quote illustrates this:

“Yes. And making sure that they [my family] are being cared for and making sure that it continues.” [1017, male, 45 years]

Also several ways of caring for household objects and assisting others were mentioned, among others being a mouthpiece to the neighbourhood, providing informal care, or assisting a child with the care for grandchildren as one participant stated:

“I have a daughter who is divorced and she has two children. When she has to work, I am there for her.” [1012, female, 69 years]

##### ICF chapter Interpersonal Interactions and Relationships

Within the ICF chapter Interpersonal Interactions and Relationships, all ICF codes and most sub-codes were mentioned (Table 2). General interpersonal interactions (d710–d729) involved having a chat and seeing people. Particular interpersonal interactions (d730–d779) involved several kinds of relationships including family relationships (d760) as well as relating with strangers (d730). An example of formal relationships (d740) is relationships with colleagues, or maintaining contacts with members of a formal association. An example of an informal social relationship (d750) is the relationship with social workers within a nursing home of a spouse, as was explained by one of the participants:

“My wife has died, and from one moment to the next, it was with the nursing home just like… All contacts were gone from one moment to te next. […] You were gone… well… finished. There is never again a talk with a social worker from that association, from that home.“[1042, male, 76 years]

Other types of relationships mentioned were family relationships (d760) and intimate relationships (d770) as was pointed out by one of the participants:

“What I want to add, intimacy and disability are quite important to me. Because initiating a personal relationship with someone also includes intimacy. And that is still underestimated too often. Whether it is only that cuddle, or real sexual intimacy, that is too rarely discussed. And that should be quite a bit more. Also with a disability, a lot is still possible.” [1005, female, 34 years]

With regard to relating with strangers (d730), one participant, for example, mentioned:

“But you ensure that you keep enough feeling with society, which already happens by walking down the street, by the way you walk down the street, the way you approach others in stores.” [1020, female, 64 years]

##### ICF chapter major life areas

Within the ICF chapter major life areas, most ICF codes and sub-codes were mentioned (Table 2). An exception was education life (d810–d839), which was not mentioned as a meaningful domain of participation in depth. None of the sub-codes within the domain of education life was mentioned by participants. Only one participant mentioned education life as a meaningful domain of participation.

With regard to work and employment (d840–d859), most ICF codes were mentioned. An illustrative quote with regard to remunerative employment (i.e. paid employment) (d850) is:

“Meanwhile I am retired. It took me some time to process it. That sounds quite heavy, but I was a German teacher for 41 years in a secondary school. That was a beautiful job, including all the stress and problems of the job of course. Because you were able to be part, also in a very modest and sometimes less modest way, of young people’s lives aged between twelve and twenty.“[1001, male, 67 years]

Also non-remunerative employment (i.e. unpaid employment) (d855) was mentioned as a meaningful domain of participation, which is illustrated by the following quote:

“I would love most of all to do paid work, but well, my body doesn’t allow that. So I fill it in in a different way, by having voluntary work and by being there for friends and acquaintances.” [1005, female, 34 years]

Some participants explicitly mentioned that remunerative employment (d850) has a different meaning to them than non-remunerative employment (d855), which is illustrated by the following quote:

“But also my work, and eventually this turns out to be the worst, since I was always busy for other people and so I did also have influence in my job. […] That’s what I miss the most. […] Participating in society… for me it’s hard to find, at least to be a satisfied participant of society when you’re actually out of society because you’ve been declared unfit.” [1029, male, 59 years]

With regard to economic life (d860–d879), basic economic transactions (d860) and economic self-sufficiency (d870) were mentioned. An illustrative quote is the following:

“Getting paid is of course actually important for very many people to be able to pay for daily business. And for their house, those kind of things.” [1025, male, 56 years]

##### ICF chapter Community, Social and Civic Life

With regard to Community, Social and Civic Life, all ICF codes, i.e. community life (d910), recreation and leisure (d920), and religion and spirituality (d930), were mentioned as meaningful participation subdomains. Community life involves both participation in formal (d9101) and informal associations (d9100), which were both mentioned by participants as meaningful subdomains of participation, while ceremonies (d902), was not mentioned. An illustrative quote with regard to participation in an informal association is:

“I organize activities for a fellow contact group. All kind of things.” [1005, female, 34 years]

With regard to recreation and leisure (d920), several kinds of leisure activities were mentioned, such as relaxing, making music, going to a party, theatre or museum (d902), engaging in sports (d901), and going on holiday. Also religion and spirituality (d930) emerged to be a meaningful participation domain among participants, which involves going to the church to profess faith as well as doing non-remunerative employment in the church which is illustrated by the following quotes:

“I go to church; I’m still active there. Yes, that also provides personal contacts, among others.” [1002, male, 78 years]

“Yes, and I would like to add something about church: I often see in surveys—I filled out more of these things—that religion is always being skipped. It seems like it was not important. While yet, for a lot of people, it is indeed important.” [1002, male, 78 years]

“I’m also a deacon at the Protestant congregation of [name of the city]. That fulfils me with satisfaction and joy, yes, to stand for something with other people.” [1001, male, 67 years]

According to the ICF conceptual framework, political life and citizenship is part of religion and spirituality. Participation in politics (d950), both passive and active, was mentioned by participants as meaningful domain of participation. Illustrative quotes are the following:

“I’m interested in political developments, so I actively follow local and worldwide news.” [1015, female, 66 years]

“I’ve been quite active in politics previously. Now, for example, I work in municipal politics.” [1045, female, 59 years]

##### Relative importance of participation subdomains

Some participants mentioned that it did not matter to them whether they participated in work, study, or in taking care of elders as long as they had at least some form of participation, which is illustrated by the following quote:

“Yes. I think that’s actually the most important thing in the participation story, that needs most attention. Yes, That can be done in many ways. It can just be a day out, but it can also be work, education. You name it.” [1004, male, 47 years]

Other participants preferred specific subdomains of participation, such as remunerative employment, above others, such as non-remunerative employment. Additionally, some participants stated that participation in one domain, for example remunerative employment, hindered participation in other subdomains, such as taking care of (grand)children. They felt forced to make choices between several subdomains of participation.

#### Part 2: Are subdomains mentioned by adults also covered by the item bank “Ability to Participate in Social Roles and Activities”?

All items of the PROMIS item bank “Ability to Participate in Social Roles and Activities” could be classified according to the ICF (sub-)codes that were assigned to the quotes of the participants. Eleven of the 50 assigned ICF sub-codes are also covered by the item bank (Table 2). But participants also mentioned participation subdomains that are currently not covered in the item bank. It concerns the following ICF codes: acquisition of necessities, education life, economic life, community life, and religion and spirituality. In addition, the items in the item bank neither distinguish between remunerative and non-remunerative employment, nor between employment and domestic life (e.g. SRPPER02r1 “I am limited in doing my work (including work at home)”), whereas this ICF distinction emerged to be important according to participants. This resulted in the situation that some items of the item bank belong to more than one ICF code, or even to more than one ICF chapter.

## Discussion

The purpose of this study was to improve measurement of participation. We found that a great variety of subdomains were mentioned as constituting to participation, which could be classified within the ICF conceptual framework. We also found that some subdomains considered important by some adults are currently not covered in the PROMIS v2.0 item bank “Ability to Participate in Social Roles and Activities”.

### Interpretation of findings

The existence of a variety of subdomains of participation is supported by previous research [15]. The subdomains that were considered meaningful depended on personal preferences, a person’s stage of life, health, and family situation. For instance, non-remunerative employment (i.e. unpaid work) represents a main activity for many individuals no longer involved in remunerative employment (i.e. paid work) because they are retired, because of health problems or for other reasons [16]. Furthermore, our study points out that specific social roles that individuals have may not be perceived as such by the individual. This was also supported by previous research [3]. To illustrate, some participants fulfilling the role of informal caregiver indicated that they did not consider this as constituting to their participation since they felt isolated and felt that this role actually hindered them in their participation. However, other participants did consider informal caregiving as constituting to their participation. Our finding that some forms of participation could hinder other forms of participation has also been reported previously. For example, a qualitative study among patients with rheumatoid arthritis regarding their experiences with participation in paid work found that some of them experienced negative interference of participation in paid work on their ability to keeping social relationships, self-care, intimate relationships with a partner, and recreation and leisure [17]. The PROMIS item bank takes into account this diversity.

Participants of the current study considered remunerative and non-remunerative employment as subdomains of participation that are fundamentally different from each other, which is in line with previous research [18]. It also becomes clear from the interviews that work at home has a different meaning compared to remunerative employment, as previously suggested [19]. However, the PROMIS item bank contains items that ask respondents about the ability to partake in work, but does not distinguish between remunerative employment, non-remunerative employment, and work at home as unique forms of participation to make sure that the items are universally applicable to people, e.g. people with and without a paid job. However, when exclusive ICF sub-codes are not measured by separate PROMIS items, the higher order ICF code might not be covered adequately. Tucker et al. suggested that ICF sub-codes need to be covered by separate PROMIS items in case sub-codes are exclusive components, such as remunerative and non-remunerative employment [9]. For example, when the ability to participate in non-remunerative employment changes, but not the ability to participate in remunerative employment, this change cannot be measured adequately [9]. As both the literature as well as adults consider remunerative and non-remunerative employment, and work at home as exclusive aspects of participation, it may be necessary to ensure that these aspects of participation are included as separate items. It may also be relevant to evaluate whether the ability to participate in remunerative employment and the ability to participate in non-remunerative employment may need to be weighted differently (in Item Response Theory terms, these sub-codes may have a different “item difficulty”).

Some subdomains (i.e. acquiring a place to live and economic self-sufficiency) were mentioned by participants as constituting to their participation. However, these are not considered as subdomains of participation by the PROMIS conceptual framework [5] but can be considered a determinant of participation. These subdomains should therefore not be part of the item bank.

### Strengths and limitations

A strength of the current study is its qualitative design, which allowed us to gain insight in the perspective of adults with regard to meaningful subdomains of participation. As our purpose is to improve the measurement of participation in the general population, the perspective of the target group is essential. Through our purposeful sampling strategy, we selected a heterogeneous sample with regard to age, sex, educational level, health problems, and meaning of participation. The advantage of a heterogeneous sample is that it contributes to evidence that the findings are not only applicable to a specific group, but to a broader range of people [20].

However, this study also has limitations. First, as participants were recruited via a national online panel of a patient organization that includes patients and caregivers who have had recent experience(s) with the healthcare system, some selection bias may have taken place. Patients who are active in an online patient panel may specifically include those who have a higher level of participation, which may influence their meaning of participation in comparison with patients who have limited ability to participate. In addition, people who have had recent experience(s) with the healthcare system may in general be older and less healthy. As a result, people with a limited ability to participate and those being young and relatively healthy may not have been included in our study. Therefore, when studying a preliminary version of the measurement instrument on participation in a large and representative sample of the Dutch population, we would recommend to add a question whether all subdomains of participation are covered. Second, there may be a downside of conducting interviews via telephone instead of face-to-face, by missing non-verbal communication cues [21]. However, as the topic was not very sensitive we assume that this did not influence the quality of our data. Third, in qualitative studies the researcher is an important instrument, both in data collection and in data analysis [22], which may have influenced the findings. We took several measures to enhance reliability of the findings. Each transcript was independently coded by two researchers and discussed afterwards. Thereafter, the findings were discussed within a group of four researchers with different backgrounds. Moreover, to ensure robustness of the findings, members of the project team—working in different fields—discussed the results. Fourth, we planned to use both deductive and inductive coding techniques, but the use of inductive coding techniques emerged not to be needed, as all participation subdomains that were meaningful to adults could be classified into the ICF conceptual framework. It might be that a different classification of subdomains of participation would have arisen, if we had started our analyses with an inductive way of coding. Finally, interview data were coded according to the ICF, and, subsequently, the items of the PROMIS item bank were classified according to the ICF and corresponding (sub) codes mentioned by adults. We have, however, not given the PROMIS item bank nor the outcome of the mapping to the participants to cross validate our classification.

### Implications for research and practice

Participation involves a diversity of subdomains, which varies between individuals and stage of life. A generic measurement instrument should take into account this diversity. The PROMIS item bank “Ability to Participate in Social Roles and Activities” offers a good starting point for the measurement of participation, as it covers items on social life, family, and work/home. However, based on the findings of the current study we recommend to explore the possibilities to extend the PROMIS item bank “Ability to Participate in Social Roles and Activities” with the following subdomains: acquisition of necessities, education life, economic life, community life, and religion and spirituality. In addition, this item bank contains items that ask respondents about the ability to partake in work, but does not distinguish between remunerative employment, non-remunerative employment, and work at home as separate subdomains of participation. As adults valued these types of work constituting differently to their participation, we recommend to consider separating these subdomains in the item bank.

While a measurement instrument for participation should take into account the diversity in subdomains of participation, such an instrument should also be relevant to everyone without being too long and time consuming. An advantage of using PROMIS item banks is that they are based on Item Response Theory, and therefore allow for selection of items that are relevant to a specific person or group (e.g. a patient group) [6]. The items can be administered as a personalized short form or through Computer Adaptive Testing, using a selected number of automatically generated items that are relevant to each specific person [6]. Because of its underlying Item Response Theory-based metric people can still be compared with each other. Therefore, we recommend to develop and validate items covering specific subdomains in which different people recognize themselves, instead of generic items. The measurement instrument should have the possibility that people decide themselves which subdomains of participation are relevant or applicable to them. This measurement instrument could then either be used for the evaluation of participation as a determinant of health or as outcome measurement of health interventions.

Measurement of participation using the PROMIS item bank “Ability to Participate in Social Roles and Activities” is also promising for clinical practice. To illustrate, at the University of Rochester Medical Center, three PROMIS instruments (Physical Function, Pain Interference, and Depression) are being completed during every outpatient clinic visit throughout 30 departments and divisions, and individual departments can choose to collect additional data relevant to their patients [23]. Research has shown that the use of PROMs in daily clinical practice increases patient–physician communication and can enhance patient care and outcomes [24]. Furthermore, the selection of items from a PROMIS item bank that are relevant to a specific person has been shown to be feasible for outcome measurement for evaluation of an intervention in a research setting [25].

## Conclusion

This study shows that participation involves many subdomains. Not all of these subdomains are currently captured in the PROMIS item bank “Ability to Participate in Social Roles and Activities”, i.e. acquisition of necessities, economic and community life, and religion and spirituality (including political life and citizenship) are missing. Also a clear distinction between paid work, volunteer work, and work at home is missing. Based on these findings, we recommend to explore the possibilities to extend the PROMIS item bank “Ability to Participate in Social Roles and Activities” with these new relevant (patient-)report areas of societal participation.

## Appendix

See Table 3.

**Table 3:** Interview guide

	Topic
Introduction	Explaining confidential treatment of interview data
Background information interviewer and purpose of the study	(1) Explaining the position of the interviewer – Researcher in the field of occupational health – Impartial and not representing a patient organization or profession (2) Introducing the topic of the interview – Participants are provided with a layman synonym of the word ‘participation’ without giving a definition – Providing a few examples of what participation could mean to someone (paid work, social activities with friends, taking care of someone who is ill) – Explaining that different people might endorse different aspects of participation – Explaining that it is unknown what adults constitute to their participation and that this information would help to develop a questionnaire that includes all aspects of participation
Main questions	(1) Background information – Level of education – Age and gender – Health problem (yes/no if yes, mental and/or physical) (2) Main questions – Meaning of participation – Satisfaction with participation – Limitations with participation (e.g. due to (a) health problem(s)) – Experiences with regard to attention for participation by healthcare professionals
End	Asking participant whether he/she has anything to add Thanking participant
